# Changing Trends in the Prevalence and Disparities of Obesity and Other Cardiovascular Disease Risk Factors in Three Racial/Ethnic Groups of USA Adults

**DOI:** 10.1155/2012/172423

**Published:** 2012-12-02

**Authors:** Camila X. Romero, Tomas E. Romero, Judith C. Shlay, Lorraine G. Ogden, Dana Dabelea

**Affiliations:** ^1^Preventive Medicine Residency Program, Colorado School of Public Health, University of Colorado Denver, Aurora, CO 80045, USA; ^2^School of Medicine, University of California, San Diego, La Jolla, CA 92093, USA; ^3^Departments of Public Health and Community Health Services, Denver Health and Hospital Authority, Denver, CO 80204, USA; ^4^Department of Family Medicine, University of Colorado Denver, Aurora, CO 80045, USA; ^5^Department of Biostatistics and Informatics, Colorado School of Public Health, University of Colorado Denver, Aurora, CO 80045, USA; ^6^Department of Epidemiology, Colorado School of Public Health, University of Colorado Denver, Aurora, CO 80045, USA

## Abstract

*Objectives*. To examine trends in the prevalence and disparities of traditional cardiovascular disease (CVD) risk factors among the major race/ethnic groups in the USA: non-Hispanic Whites (NHWs), non-Hispanic Blacks (NHBs), and Mexican Americans (MAs). *Methods*. We used cross-sectional trend analysis in women and men aged 25–84 years participating in the NHANES surveys, years 1988–1994 (*n* = 14,341) and 1999–2004 (*n* = 12,360). *Results*. The prevalence of obesity and hypertension increased significantly in NHW and NHB, both in men and women; NHB had the highest prevalence of obesity and hypertension in each time period. Diabetes prevalence showed a nonsignificant increasing trend in all groups and was higher in MA in both periods. Smoking significantly decreased in NHW men and NHB, the latter with the largest decline although the highest prevalence in each period; no changes were noted in MA, who had the lowest prevalence in both periods. Race/ethnic CVD risk factors disparities widened for obesity and hypercholesterolemia, remained unchanged for diabetes and hypertension, and narrowed for smoking. *Conclusions*. The increasing prevalence of obesity and hypertension underscores the need for better preventive measures, particularly in the NHB group that exhibits the worst trends. The decline in smoking rates may offset some of these unfavorable trends.

## 1. Introduction

It has been of note that despite the increasing obesity prevalence in the USA observed in previous decades, a decline in CVD risk factors has occurred which has been attributed to the influence of lifestyle changes and therapies [1–3]. However, in the last decade, CVD risk factor downward trends seem to have reached a plateau [1, 2] or even undergone the beginning of an unfavorable increase [2]. 

Many studies have documented race/ethnic differences in the prevalence of all major CVD risk factors [4–10]; a higher prevalence of hypertension in African Americans and diabetes in Hispanics are some well-known examples although limited information has been published on race/ethnic CVD risk factors trends over time. It was only since 1988 when the National Health and Nutrition Examination Survey (NHANES) oversampled underrepresented race/ethnic groups, that accurate national estimates of Mexican and African Americans were available for race/ethnic-specific analyses. One of the overarching goals of the Healthy People 2010 initiative is to eliminate health disparities among USA race/ethnic groups. A better understanding of whether trends in the prevalence of obesity and other CVD risk factors follow a similar or different pattern across USA race/ethnic groups represents an essential step in our progress towards meeting this goal. Therefore, the main objectives of this study were to examine trends in the prevalence of obesity along with smoking, diabetes, hypertension, and hypercholesterolemia among the three major race/ethnic groups in the USA, non-Hispanic White, non-Hispanic Black, and Mexican-Americans, and to test whether the prevalence and trends differ according to race/ethnicity. 

## 2. Methods

### 2.1. Study Population

Data from two NHANESs conducted from 1988 to 2004 (NHANES III: 1988–1994 and NHANES 1999–2004) were used. NHANESs are a set of large national cross-sectional surveys of the USA civilian, noninstitutionalized population based on interview, examination, and laboratory information. Detailed descriptions of the plan and operation of each survey have been published [11–14]. Beginning in 1999, NHANES was converted from a static survey to a continuous annual survey providing periodic data releases. The NHANES data releases for 1999-2000, 2001-2002, and 2003-2004 were aggregated into a combined dataset (NHANES 1999–2004) to enable comparability with the 6-year NHANES III. Each of the surveys followed a stratified, multistage probability design in which a sample of the USA population was selected. Both NHANES III and NHANES 1999–2004 oversampled Mexican and African American populations in order for the sample to produce statistically reliable health estimates for these groups.

NHANES data for both survey periods were collected via standardized questionnaires administered by bilingual interviewers at participants' homes, standardized examinations, and nonfasting laboratory tests conducted and collected by health examiners at NHANES mobile examination centers. NHANES III and NHANES 1999–2004 underwent institutional review board approval and included written informed consent. The sample for this analysis included women and men aged 25–84 years who completed both a home-based questionnaire and the medical examination. The overall response rates for both the questionnaire and examination and the sample populations for NHANES III and NHANES 1999–2004 were 73.4% (14,341 persons) and 72.2% (12,360 persons), respectively.

### 2.2. Definition of Variables

Race and ethnicity were self-reported using the categories of Black, White, or other (the latter category was created by combining three other race categories) for race, and non-Hispanic, Mexican American, or other Hispanic for ethnicity. Four race/ethnic groups were defined for both survey periods: non-Hispanic White (NHW), non-Hispanic Black (NHB), Mexican American (MA), and other race/ethnicity. The first three race/ethnic groups were used for analysis. The sample sizes for the 1988–1994 and 1999–2004 study periods for NHW were 6,028 and 6,253, NHB were 4,009 and 2,434, and MA were 3,734 and 2,766, respectively. 

### 2.3. Definition of CVD Risk Factors

The five CVD risk factors in this study were measured using similar methods in the two survey periods, as defined in previous publications [1, 3]. The laboratory methods used at the mobile examination center have been reported in detail elsewhere [15]. The five CVD risk factors were defined and measured as follows. 

Obesity was defined as body mass index (BMI) ≥30 kg/m^2^. Height and weight were obtained using standard protocols [12–14]. 

Current smoking status was determined based on positive answers to the following questions asked during the household survey: “Have you smoked at least 100 cigarettes in your life?” and “Do you smoke cigarettes now?” 

Diabetes status was defined as the presence of a fasting plasma glucose ≥126 mg/dL or self-reported current use of a glucose-lowering medication (insulin or hypoglycemic medications). Blood samples for fasting plasma glucose were assessed using previously described procedures for blood collection and processes [11]. Diabetes prevalence estimates were calculated from an NHANES subpopulation of individuals who participated in the home questionnaire, medical examination, and in the morning fasting laboratory exam (period I: 7,117 and period II: 6,069 persons). 

Hypertension was defined as systolic blood pressure (SBP) ≥140 mm Hg or diastolic blood pressure (DBP) ≥90 mm Hg or self-reported current use of antihypertensive medication. Blood pressure was measured according to standard protocols [12–14]. 

Hypercholesterolemia was defined as total cholesterol levels ≥200 mg/dL or self-reported use of lipid-lowering medication. Total cholesterol concentrations were collected during the medical examination and processed according to criteria of the CDC-National Heart, Lung and Blood Institute Lipid Standardization Program.

### 2.4. Data Analysis

Statistical analyses were performed using SAS version 9.1 (SAS Institute Inc., Cary, NC, USA) statistical software. The estimated means and standard errors were computed using the procedures SURVEYFREQ, SURVEYMEANS, and SURVEYREG [16]. These procedures accounted for the complex NHANES multistage stratified cluster sample design and allowed for sample weights to adjust for the unequal probabilities of selection, oversampling, and nonresponse. Prevalence estimates of five CVD risk factors (current smoking, diabetes, hypertension, hypercholesterolemia, and obesity) and mean values of SBP, DBP, BMI, and total cholesterol were compared across two NHANES time periods: period I (1988–1994) and period II (1999–2004) and three race/ethnic groups (NHW, NHB, and MA). To allow for comparisons between race/ethnicities and across time, prevalence and mean estimates were age-adjusted to the 2000 Census Population. Absolute change in age-adjusted prevalence estimates for each risk factor within each race/ethnic group over time and the difference in absolute change between race/ethnic groups for each period and over time were calculated by the two-sided Wald test at the 0.05 significance level. Unequal variances were assumed for the two time points; therefore, the Welch-Satterthwaite method was used to approximate degrees of freedom [16]. 

## 3. Results

The distribution of age groups, sex, and education categories were generally similar among the NHW, NHB, and MA populations over the two survey periods: period I (1988–1994) and period II (1999–2004). There were some exceptions: education levels across all race/ethnic groups increased over time, and the proportion of younger (age 25–34 years) individuals decreased, while the proportions of those aged 45–54 and 75–84 increased over time. MA had increased proportions of younger individuals; both NHB and MA populations had greater proportions of less educated participants, compared with the NHW population (Table 1). 

### 3.1. Obesity

The prevalence of obesity significantly increased from period I to period II: NHW (22.8% to 30.8%, *P* < 0.0001); NHB (32% to 42.4%, *P* < 0.0001) (Figure 4(a)), involving in a similar fashion men and women (Table 2), although in MA the increase in prevalence was observed only in men and reached borderline significance (31.5% to 35.1%, *P* = 0.06) (Table 2). The largest increases in prevalence occurred in NHB women (40.3% to 52%, *P* < 0.0001). NHB women, followed by MA women, had the highest prevalence in 1999–2004 (52% and 40.6%, resp.) (Table 2, Figure 3). NHW versus NHB and NHW versus MA differences were significant in period I, with a significant difference seen in period II between NHB versus MA (*P* = 0.007). These race/ethnic disparities widened between NHB and MA but stayed mostly the same between the other race/ethnicities. (Figure 4(b)).

### 3.2. Current Smoking

**Figure 1:**
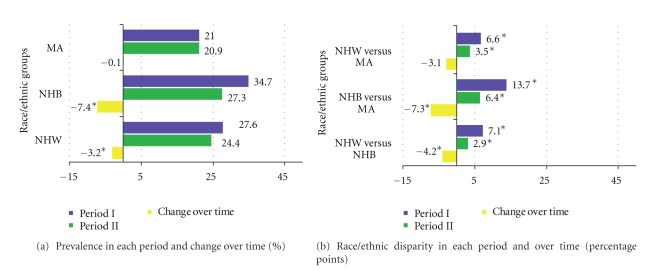
Current smoking prevalence and race/ethnic disparity in each period and over time; NHANES periods I (1988–1994) and II (1999–2004).

Prevalence of current smoking decreased significantly from period I to period II for NHW (27.6% to 24.4%, *P* = 0.02) and NHB (34.7% to 27.3%, *P* < 0.0001) but did not change in MA (21% to 20.9%, *P* = 0.96) which had the lowest prevalence in both periods (Table 2) (Figure 1(a)). The largest decrease occurred among NHB men (−8.4 percentage points, *P* = 0.0003); NHW men and NHB women also showed a significant decrease (Table 2); however, NHB men still had the highest prevalence of current smoking in 1999–2004. All the race/ethnic group differences in prevalence estimates for periods I and II were statistically significant. The race/ethnic differences significantly narrowed over time between NHW versus NHB (*P* = 0.05) and NHB versus MA (*P* = 0.0006), mostly due to NHB decreases in prevalence estimates over time. (Figure 1(b)).

### 3.3. Diabetes

**Figure 2:**
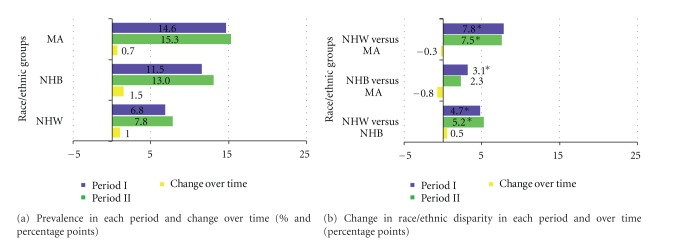
Diabetes prevalence and race/ethnic disparity in each period and over time; NHANES periods I (1988–1994) and II (1999–2004).

Prevalence of diabetes nonsignificantly increased over time for all race/ethnic groups from period I to period II: NHW (6.8% to 7.8%); NHB (11.5% to 13%); MA (14.6% to 15.3%). (Table 2 and Figure 2(a)) MA men and women had the highest prevalence of diabetes in both periods (15.4% and 15%, resp.), as shown in Table 2. No significant gender differences in diabetes prevalence were observed in the three race/ethnic groups although NHW men trended to a higher prevalence than NHW women in both periods (Table 2). All the race/ethnic group differences in prevalence estimates were statistically significant in period I; however, in period II, the NHB versus MA difference was no longer statistically significant, mostly due to the prevalence of diabetes trending upward more so in NHB compared to MA. There were no significant changes in race/ethnic disparities over time (Figure 2(b)).

### 3.4. Hypertension

**Figure 3:**
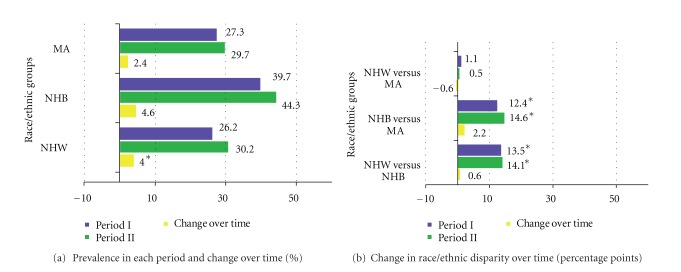
Hypertension prevalence and change in race/ethnic disparity in each period and over time; NHANES periods I (1988–1994) and II (1999–2004).

**Figure 4:**
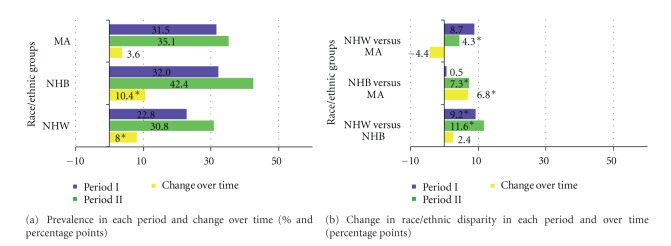
Obesity prevalence and race/ethnic disparity in each period and over time; NHANES period I (1988–1994) and period II (1999–2004).

Prevalence of hypertension significantly increased from period I to period II, NHW (26.2% to 30.2%, *P* = 0.0006); NHB (39.7% to 44.3%, *P* = 0.001), and increased nonsignificantly among MA (27.3% to 29.7%, *P* = 0.1). (Table 2, Figure 3(a)). The largest increase occurred in women (NHW, NHB, and MA) and to a lesser extent in men, with the exception of MA. NHB women and men had the highest prevalence of hypertension in 1999–2004 (45.6% and 42.5%, resp.) (Table 2). NHW versus NHB and NHB versus MA differences in prevalence estimates for Periods I and II were statistically significant. These race/ethnic disparities did not change significantly over time (Figure 3(b)).

### 3.5. Hypercholesterolemia

The prevalence of hypercholesterolemia decreased significantly from period I to period II in NHB (54.2% to 50.5%, *P* = 0.01), a change that was driven by the large decrease observed in NHB women but did not change significantly in NHW (57.5% to 59.2%, *P* = 0.2) and in MA (57.3% to 55.8%, *P* = 0.4) populations (Table 2). Differences in prevalence estimates were statistically significant between NHB and the other race/ethnic groups in period I and in period II; these race/ethnic disparities widened mostly due to greater decreases in NHB over time. A significant race/ethnic disparity between NHW and MA emerged due to the trending downward prevalence estimate in the MA group. 

## 4. Discussion

This study documents a significant increase in the prevalence of obesity and hypertension between 1988 and 2004 in NHW and NHB, with an increasing trend observed in MA. Also, an increasing trend (nonsignificant) in the prevalence of diabetes over time in all race/ethnic groups was found. This is consistent with the increasing prevalence of obesity and suggests that, in the future, unless effective methods to halt the obesity epidemic are implemented, the prevalence of diabetes will likely augment. Increases in diabetes prevalence have been reported in the last decade, which has disproportionately affected MA and NHB populations [2, 17, 18]. 

The finding that the prevalence of current smoking for most race/ethnic groups significantly decreased is encouraging, as is the narrowing of race/ethnic disparities in the prevalence of smoking over time. However, NHB still has the highest prevalence of current smoking and race/ethnic disparities persisted in 1999–2004; therefore, continuedpublic health efforts are needed to target this population, especially NHB men. 

In this study, the prevalence of hypercholesterolemia significantly decreased for NHB and trended downward for MA and upward for NHW despite an increase in obesity prevalence. While this may appear paradoxical, previous studies have suggested that prevalence of high cholesterol and an elevated BMI are not necessarily related [19]. Furthermore, our study found a widening race/ethnic disparity between NHW and NHB for hypercholesterolemia, which was primarily driven by opposite trends in these race/ethnic groups. 

The increase in obesity prevalence among USA adults over time has been reported previously [3, 20]. In our study, it increased significantly from period I to period II in both genders and in MA men but not in MA women. A 1999–2004 trend analysis found no race/ethnic differences in obesity prevalence in men; however, NHB and MA women were significantly more likely to be obese compared with NHW women [6]. Our study corroborates these findings in sex-stratified analyses. Furthermore, we found that race/ethnic disparities in obesity prevalence widened between NHB and MA, mostly due to greater increases over time in NHB women as compared to their MA counterparts. A recently published study analyzing NHANES data from 1999 to 2008 found a significant increasing obesity trend in men (4.7%) but not in women (2.1%), although it confirmed the higher obesity prevalence in NHB and MA women (49.6% and 45.1%, resp.) in contrast to NHW women (33%) [21]. These values are similar to the prevalence found in our study for period II (1999–2004).

Growing trends in obesity prevalence may lead to increases in diabetes and hypertension as previously reported in other studies [7, 19], with our data suggesting that these worsening trends are already occurring. A recently released report by the Center for Disease Control and Prevention (CDC) analyzing NHANES data from 1999 to 2008 indicated an increase of hypertension prevalence from 28.1% to 30.9% [22, 23]. The higher prevalence of obesity in NHB and MA as compared to NHW underscores the influence of socioeconomic factors reported in previous studies [1, 4, 24–26]. We also found NHB and MA groups to be more represented in the lower-education group in both periods. 

Although obesity prevention should be the primary public health focus in order to address future worsening trends in diabetes and hypertension, there are multiple challenging factors involved in a population-oriented strategy to address these trends. Observational studies and a randomized long-term social experiment recently published have shown the impact of neighborhood characteristics and built environment in the prevalence of obesity and diabetes [27–29]. Increasing opportunities provided by better neighborhood attributes to access educational facilities, health clinics, healthier food outlets, green areas, and public parks with less crime have been suggested as determining factors. However, reservations regarding the significance of BMI as a predictor of health outcomes and survival have been published, with data indicating that higher mortality is definitively associated with BMI > 35 kg/m^2^, but not clearly demonstrated in the overweight group, especially those in the BMI 25–30 kg/m^2^ range [30]. Moreover, increasing relevance has been given in the last decade to the abdominal distribution of fat in the prediction of cardiovascular risk, and its measurement is becoming a standard method of risk assessment [31–33]. 

The relationship between lifestyle-related behaviors, such as increased physical activity and healthful dietary habits, with obesity prevention and lowering of CVD risk is well known [18, 21]. Recent self-report data from a 2003 national cross-sectional survey found race/ethnic differences in lifestyle behaviors: NHB and MA adults as compared to NHW had a lower prevalence of physical activity and consumption of 5 or more servings of fruits and vegetables [10]. As previously discussed, these limitations may be related to socioeconomic factors determining the neighborhood characteristics where NHB and MA live. A 1988–1994 NHANES study of adult USA women found that CVD risk factors were higher in MA and NHB compared with NHW women, and that after adjusting for years of education, significant differences in blood pressure, BMI, physical inactivity, and diabetes remained [4]. Our data indicate that racial/ethnic differences have persisted or even worsened since 1998–1994. Considering that race/ethnic minorities (NHB and MA) usually have more unfavorable CVD risk factor profiles than NHW, the persistence of race/ethnic disparities for smoking, diabetes, and hypertension and the widening of disparity for obesity over time suggest the need for targeting efforts for these race/ethnic groups. 

Our study has several limitations. First, race and ethnicity disparities research is a relatively burgeoning field, and controversy exists as to the interpretation of differences based on race and ethnicity data alone [34–36]. However, NHANES uses standard methodology and since 1988 is the most accurate source for US national estimates for underrepresented minorities. Second, socioeconomic features that are associated to the prevalence of CVD risk factors and race/ethnicity characteristics were not evaluated as confounders as this was a descriptive study. Third, CVD risk factors were based on self-report data, which may result in misclassification. However, previous studies using NHANES data have validated self-reported smoking with cotinine [37] and found low levels of underreporting. Misclassification could also have occurred if participants provided inaccurate information on treatment for hypertension, hypercholesterolemia, or diabetes. In addition, treatment was narrowly defined as the use of medications, since other treatment methods, such as lifestyle modifications (weight loss and exercise), were not collected. Temporal changes in the prevalence of hypertension, and hypercholesterolemia may have been influenced by changes in treatment patterns for these conditions over time. Fourth, important CVD risk factors were not included in our study. For example, physical activity was not included because NHANES lacked consistency in the types of physical activity questions collected over time. Finally, laboratory measures were based on one blood sample, and several blood pressure measurements were taken on one day only, which may overestimate prevalence of hypertension or hypercholesterolemia. 

Nevertheless, our study is based on comprehensive national surveys for USA adults, including oversampling of NHB and MA individuals. Only since the release of 2002–2004 NHANES survey could accurate and nationally representative estimates among race/ethnic groups be used to examine changes over time for 10-year trend analysis. 

## 5. Conclusions

Important temporal trends in the prevalence of major CVD risk factors in a representative sample of USA NHW, NHB, and MA adults were documented: a worrisome increase in the prevalence of obesity and hypertension in most groups and a decrease in the prevalence of current smoking. To what extent these unfavorable CVD risk factors trends may be offset by the decline in smoking is unknown. This study also found persistent race/ethnic differences for all CVD risk factors and time periods examined, with NHB and MA generally having worse profiles than NHW. Importantly, race/ethnic disparities did not improve over time (with the exception of smoking); in fact, they widened for obesity and hypercholesterolemia. Disproportionate increases in the prevalence of obesity may be implicated in persistent disparities observed for hypertension and diabetes currently and in the future. Furthermore, recent data suggest that the decline in CVD mortality observed in the last 50 years may be leveling off due to alarming increases in the prevalence of modifiable CVD risk factors [38, 39]. Our results highlight the need to increase cardiovascular primary prevention, particularly obesity prevention, among all race/ethnic groups. 

## Figures and Tables

**Table 1:** Demographic characteristics among NHW, NHB, and MA adults, by period I (1988–1994) and period II (1999–2004).

		Non-Hispanic White		Non-Hispanic Black		Mexican American	
		NH 88–94 (*n* = 6,028)	NH 99–04 (*n* = 6,253)	NH 88–94 (*n* = 4,009)	NH 99–04 (*n* = 2,434)	NH 88–94 (*n* = 3,734)	NH 99–04 (*n* = 2,766)
Sex	Male	48.3	48.4	44.5	43.9	51.6	53.1
	Female	51.7	51.6	55.5	56.1	48.4	46.9

Age, years	25–34	25.6	19.2	32.1	24.6	39.8	37.3
	35–44	25.0	23.7	29.0	27.5	28.5	29.1
	45–54	16.7	23.0	13.8	22.2	15.2	17.5
	55–64	14.0	14.5	12.2	13.3	9.0	8.6
	65–74	12.4	11.7	8.8	8.2	5.7	5.4
	75–84	6.2	7.9	4.1	4.3	1.7	2.1

Education	<HS	20.1	13.8	32.9	33.4	59.5	55.0
	HS	34.8	27.6	36.9	24.4	22.2	19.6
	>HS	45.1	58.6	30.2	42.2	18.3	25.4

Values are percentages.

NH: NHANES, HS: high school.

**Table 2:** Changes over time in age-adjusted prevalence of CVD risk factors among NHW, NHB, and MA adults, by sex and period I (1988–1994) and period II (1999–2004).

	Current Smoking			Diabetes			Hypertension			Hypercholesterolemia			Obesity		
	I	II	Δ	I	II	Δ	I	II	Δ	I	II	Δ	I	II	Δ
Non-Hispanic White															

Men	29.5	25.6	−3.9 *	8.4	10.5	2.1	27.5	30.6	3.1 *	58.3	59.5	*1.2 *	21.3	29.7	8.4 **
Women	25.9	23.3	*−2.6 *	5.4	5.2	−0.2	24.5	29.3	4.8 **	56.2	58.4	*2.2 *	24.2	31.9	7.7 **

Total	**27.6 **	**24.4**	−3.2 *	**6.8**	**7.8**	**1.0**	**26.2**	**30.2**	4.0 **	**57.5**	***59.2 ***	***1.7 ***	**22.8**	**30.8**	8.0 **

Non-Hispanic Black															

Men	42.3	33.9	−8.4 **	10.3	11.5	1.3	38.8	42.5	3.7 *	51.6	51.1	*0.5 *	21.2	30.1	8.9 **
Women	28.8	22.1	−6.6 *	12.3	13.9	1.6	40.3	45.6	5.3 *	56.0	50.0	−5.9 *	40.3	52.0	11.7 **

Total	**34.7 **	**27.3**	−7.4 **	**11.5**	**13.0**	**1.5**	**39.7**	**44.3**	4.6 *	**54.2**	***50.5 ***	−3.7 *	**32.0**	**42.4**	10.4 **

Mexican-American															

Men	27.6	27.4	*−0.2 *	13.7	15.4	1.7	28.1	28.2	*0.1 *	58.5	56.9	*−1.6 *	25.7	30.0	4.3 *
Women	14.0	13.8	*−0.2 *	15.3	15.0	−0.3	26.3	30.9	4.6 *	55.6	54.3	*−1.3 *	37.5	40.6	*3.1 *

Total	**21.0 **	**20.9**	***−0.1 ***	**14.6**	**15.3**	**0.7**	**27.3**	**29.7**	***2.4 ***	**57.3**	***55.8 ***	***−1.5 ***	**31.5**	**35.1**	***3.6 ***

Values are percentages, except that the change column values are percentage points.

I = NHANES 1988–1994; II= NHANES 1999–2004.

The asterisks (* and **) indicate significant changes overtime, *P* value < 0.05* and < 0.001**, respectively.

Δ = change.
